# Identification and Characterization of MicroRNAs from Tree Peony (*Paeonia ostii*) and Their Response to Copper Stress

**DOI:** 10.1371/journal.pone.0117584

**Published:** 2015-02-06

**Authors:** Qijiang Jin, Zeyun Xue, Chunlan Dong, Yanjie Wang, Lingling Chu, Yingchun Xu

**Affiliations:** College of Horticulture, Nanjing Agricultural University, Nanjing, 210095, China; Kunming University of Science and Technology, CHINA

## Abstract

MicroRNAs (miRNAs) are a class of non-coding, small RNAs recognized as important regulators of gene expression. Although plant miRNAs have been extensively studied in model systems, less is known in other plants with limited genome sequence data, including *Paeonia ostii*. In this work, we used high-throughput sequencing to identify conserved and nonconserved miRNAs and other short RNAs in *Paeonia ostii* under control and copper stressed condition. 102 previously known plant miRNAs were identified and classified into 89 families according to their gene sequence identity. Some miRNAs were highly conserved in the plant kingdom suggesting that these miRNA play important and conserved roles. Combined our transcriptome sequencing data of *Paeonia ostii* under same conditions, 34 novel potential miRNAs were identified. The potential targets of the identified known and novel miRNAs were also predicted based on sequence homology search. Comparing the two libraries, it was observed that 12 conserved miRNAs and 18 novel miRNAs showed significantly changes in response to copper stress. Some of the new identified potential miRNAs might be involved in *Paeonia ostii*-specific regulating mechanisms under copper stress. These results provide a framework for further analysis of miRNAs and their role in regulating *Paeonia ostii* response to copper stress.

## Introduction

Heavy metal pollution is a major environmental problem facing the modern world. Essential heavy metals such as copper and zinc are necessary for a wide range of physiological processes [1]. Copper, for example, serves as a vital component of electron-transfer reactions mediated by proteins. However, elevated concentrations of essential as well as non-essential heavy metals can result in growth inhibition and toxicity symptoms of most plants [2]. Some non-essential metals (e.g. cadmium) are potentially highly toxic even at low concentrations [3]. The characteristic feature of toxicity symptoms in the presence of excessive amounts of heavy metals is the inhibition or disruption of enzyme systems and the induction of oxidative stress [2,4]. In order to maintain the correct concentrations of essential metal ions and to minimize the damage from exposure to hazardous metal ions, plants have evolved a complex network of homeostatic mechanisms to control the uptake, accumulation, trafficking and detoxification of metals. For the need of agricultural practice and phytoremediation of metal-contaminated soils, a variety of studies have been made to investigate the mechanisms of plant metal homeostasis and tolerance at physiological, biochemical, and molecular levels [5–7]. The best described mechanism involves the intracellular metal chelation by a series of Cys-rich peptides such as glutathione (GSH), phytochelatins (PCs) and metallothioneins (MT) [8,9]. Additionally, a number of genes encoding specific membrane transport proteins such as ATP-binding cassette (ABC) transporters, cation diffusion facilitators (CDF) and copper transporters (COPT) have been found to mediate heavy metal uptake and transport in plant [10–12]. Metal transport, chelation and sequestration formed a synergic network to maintain the proper delivery and distribution of metal ions in plant cell, resulting in a basic level of metal tolerance [5–7]. Loss of one of these critical processes leads to plant hypersensitivity. Transcriptional and post-transcriptional gene regulation is a key step in this network for the response to metal exposure or metal deficiency. However, the gene regulatory mechanism in plant metal homeostasis is largely unknown [2].

Recently, growing evidence has revealed that microRNAs (miRNAs) function as key regulators in the alleviation of plant metal stresses [2,13–15]. miRNAs are a recently discovered class of small (19–24 nucleotides in length), non-coding RNAs that regulate mRNA or protein level either by promoting mRNA degradation or by attenuating protein translation [16]. The role of plant miRNAs was initially reported to be involved in a wide range of biological processes such as cell cycle control, apoptosis and several developmental and physiological processes [16,17]. Recently, increasing evidences indicate that miRNAs also play critical roles in regulating plant response to biotic and abiotic stress including heavy metals stress [18–20]. For example, Osa-miR604 functions in the regulation of Cd tolerance through directing degradation of lipid transfer protein (LTP) mRNA [21]; Several works suggest a strong link between miR398, miR397, miR408, and miR857 and Cu homeostasis [22]. Although some miRNAs have been well elucidated in previous studies, more miRNAs were supposed to participate in plant response to heavy metal stress. Thus, large-scale detailed analysis of miRNAs and their targets involved in heavy metal stress may provide a new insight into understanding of plant heavy metal stress response mechanisms.

*Paeonia ostii* is one of the most important horticultural crops in the world due to its striking ornamental and medicinal values. *Paeonia ostii* belongs to the Moutan subfamily which comprises eight wild species including *Paeonia cathayana*, *Paeonia jishanensis*, *Paeonia qiui*, *Paeonia ostii*, *Paeonia rockii*, *Paeonia decomposita*, *Paeonia delavayi* and *Paeonia ludlowii* [23,24]. Among all the wild species, *Paeonia ostii* was proved to be highly tolerant to copper stress. As a wild species and its close relation with other cultivars, *Paeonia ostii* can be a preferred candidate to perform sRNA sequencing and analyze their role in copper stress response. However, no systematic studies of small RNA in *Paeonia ostii* have been conducted. The focus of this work is to analyze miRNAs as well as their targets involved in *Paeonia ostii* Cu homeostasis and tolerance. It will extend the current view on the molecular understanding of miRNA-guided regulation of plant heavy metal adaption.

## Materials and Methods

### Plant materials, growth conditions and Cu treatment

*Paeonia ostii* (Paoenia ostii ‘Feng Dan’) was used in this study. *Paeonia ostii* seedlings were cultured in nutrient medium (quarter-strength MS solution) at 20/15°C (day/night), with a light intensity of 200 μmol m^-2^s^-1^ and 12 h photoperiod. After growing for 1 week, uniform seedlings were incubated in solution containing 0 and 100 μM CuSO_4_ for different times. For small RNA sequencing, the roots, stems and leaves of seedlings were separately harvested after 1, 6, 12 and 24 h of treatment and immediately frozen in liquid nitrogen for analysis.

### Small RNA library preparation and sequencing

Total RNA was extracted from frozen roots, stems and leaves of *Paeonia ostii* with Trizol (Invitrogen, Carlsbad, CA, USA). Two sets of total RNA were prepared, with one derived from the original RNA pool prepared form Cu-treated tissues (roots, stems and leaves) (+Cu) at 1, 6, 12 and 24 h time points and the other form the RNA pool derived from Cu-free tissues (-Cu) at the same time points. The samples were then subjected to 15% denaturing polyacrylamide gel electrophoresis, after which the sRNA fragments of 18–28 nt were isolated from the gel and purified. Next, the sRNA molecules were ligated to a 5’ adaptor and a 3’ adaptor sequentially and then converted to DNA by RT-PCR. Finally, approximately 20 μg products of RT-PCR was sequenced directly using Solexa 1G Genome Analyzer according to the manufacturer’s protocols (BGI, Shenzhen, China). Sequence data from this article can be found in the GEO database of NCBI under accession numbers GSE62661.

### Small RNA analysis

Clean reads were screened from raw sequencing reads by removing contaminated reads including sequences with 5’-primer contaminants, without the inserted tag, with poly(A) tails, either shorter than 15 nt or longer than 30 nt. After removing the adaptor/acceptor sequences, filtering the low quality tags and cleaning up the contamination formed by the adaptor-adaptor ligation, the occurrences of each unique sequence read were counted as sequence tags. The remaining unique RNAs were mapped to the *Paeonia ostii* transcriptome sequencing data (Wang et al, unpublished data; Gai et al., 2013) using SOAP 2.0 program [25,26]. Sequences with a perfect match were retained for further analysis. All these sequence tags were compared with the sequences of non-coding RNAs (rRNA, tRNA, snRNA, snoRNA) available in Rfam (http://www.sanger.ac.uk/software/Rfam) [27] and the GenBank noncoding RNA database (http://www.ncbi.nlm.nih.gov/) to classify degradation fragments of noncoding RNA. The rest of the sequences which could match *Paeonia ostii* transcriptome sequencing data were searched for miRNA sequences using miRBase 20 (http://www.mirbase.org/index.shtml) [28]. To annotate the miRNAs in *Paeonia ostii*, the following processes were performed: (1) considering the difference among species, the clean data were first aligned to the miRNA precursor/mature miRNA of all plants in miRBase allowing two mismatches and free gaps to get temporary miRNA sequences and count of miRNA families (no specific species); (2) choose the highest expression miRNA for each temporary mature miRNA family to form a temporary miRNA database of *Paeonia ostii*; (3) align clean data to the above temporary miRNA database to identify conserved miRNAs in *Paeonia ostii*, in which only perfectly matched or closely related (allowing up to two mismatches) sequences were considered conserved miRNAs. Based on the identified conserved miRNAs of *Paeonia ostii*, we performed extensive comparisons against known miRNAs in other plant species to investigate the evolutionary conservation relationship of known miRNAs in *Paeonia ostii* and other plants.

### Prediction of novel miRNA

Prediction of *Paeonia ostii* miRNAs was conducted using criteria that were previously developed for plant miRNA prediction [29]. MiRNA precursors have characteristic fold-back structures that can be used to predict novel miRNAs. The prediction was implemented in the MIREAP program developed by the BGI (https://sourceforge.net/projects/mireap/). Some key conditions are as following: 1. The tags used to predict novel miRNA were from the unannotated tags in clean data; 2. The pre-miRNA of predicted mature miRNA could form an appropriate stem-loop structure with a mature miRNA sitting in one arm of the hairpin structure; 3. The secondary structures of the hairpins are steady, with the free energy of hybridization lower than or equal to-18 kcal/mol; 4. Hairpin precursors lack large internal loops or bulges; 5. The mature miRNA strand and its complementary strand present 2-nucleotide 3′ overhangs; 6. The reads of predicted mature miRNA must more than 5 [30]. The reads of novel miRNA is calculated by summing small RNAs which could perfectly match or closely related (allowing up to three mismatches in the 5′ or 3′ end) to mature miRNA.

### Prediction of miRNA targets

The identified known miRNAs and predicted novel miRNAs were used to interrogate sequences for target sites on the psRNAtarget web server (http://biocomp5.noble.org/psRNATarget/) using *Paeonia ostii* transcriptome sequencing data. The target transcripts containing complementary sequences of miRNAs were determined with previously established criteria [31–33]. The criteria for target prediction are as following: (1) No more than four mismatches between sRNA and target (G-U bases count as 0.5 mismatches); (2) No more than two adjacent mismatches in the miRNA/target duplex; (3) No adjacent mismatches in positions 2–12 of the miRNA/target duplex (5′ of miRNA); (4) No mismatches in positions 10–11 of miRNA/target duplex; (5) No more than 2.5 mismatches in positions 1–12 of the of the miRNA/target duplex (5′ of miRNA); (6) Minimum free energy (MFE) of the miRNA/target duplex should be > = 75% of the MFE of the miRNA bound to it’s perfect complement. The functional category of obtained target sequences was annotated against the COG database (http://www.ncbi.nih.gov/COG/) using BLAST program with a cutoff of E value <1e-5.

### Differential expression analysis of miRNAs under copper stress

To investigate the differentially expressed miRNAs between libraries, we compared the gene expression patterns of miRNAs in CK and TR library. Towards this purpose, we considered the following criteria: (1) adjusted *P*-value should be less than 0.01 (*P*-value<0.01) in at least one data set. (2) fold change or log_2_ ratio of normalized counts between CK and TR libraries was greater than 1 or less than-1 in one of the libraries. The frequency of miRNA read counts was normalized as transcripts per million (TPM) and normalization of miRNA expression levels between CK and TR was carried out based on the following formula: Normalization formula: (*Actual miRNA count* / *Total count of clean reads*)*10^6^

Afterwards, the fold-change between treatment and control and *P*-value were calculated from the normalized expression using the formula shown below:

Fold-change formula: Fold change = log_2_(*treatment*/*control*)

*P*-value formula:

(xy)=(N2N1)Y(x+y)!x!y!(1+N2N1)(x+y+1)D(y≥ymax|x)=∑y≥ymax∞p(y/x)C(y≤ymin|x)=∑y=0y≤yminp(y/x)

where N_1_ and N_2_ are sampling size (total reads of two libraries respectively), x and y are read of specific miRNA in two libraries respectively. To compute the confidence intervals, we made use of the cumulative distributions:$C\left(y\leq y_{\min}|x\right)=\sum_{y=0}^{y\leq y_{\min}}p(y/x)$ and $D\left(y\geq y_{\max}|x\right)=\sum_{y\geq y_{\max}}^{\infty }p(y/x)$ which allow the computation of an interval [y_min_, y_max_]_ε_ and serve as a significance test when comparing [34].

Poisson distribution model was used for estimating the statistical significance of miRNA expression changes under control and treatment conditions [34]. Up-regulation of any miRNA expression levels was considered a positive value while negative values indicate down-regulation.

### Confirmation of mature miRNAs expression

For determination of miRNA expression, RNAs were reverse-transcribed by One Step PrimeScript miRNA cDNA Synthesis Kit (TaKaRa), which added a ploy (A) tail to the 3’-end of miRNA and with transcription leading by a known oligo-dT ligate. SYBR Premix Ex Tag II (TaKaRa) was used for qRT-PCR. Small nuclear RNA U6 was used as an internal reference. Real-time quantitative RT-PCR reactions were performed using a real-time PCR system (Eppendorf, Hamburg, Germany).

## Results

### High-throughput sequencing of small RNAs

**Fig 1:**
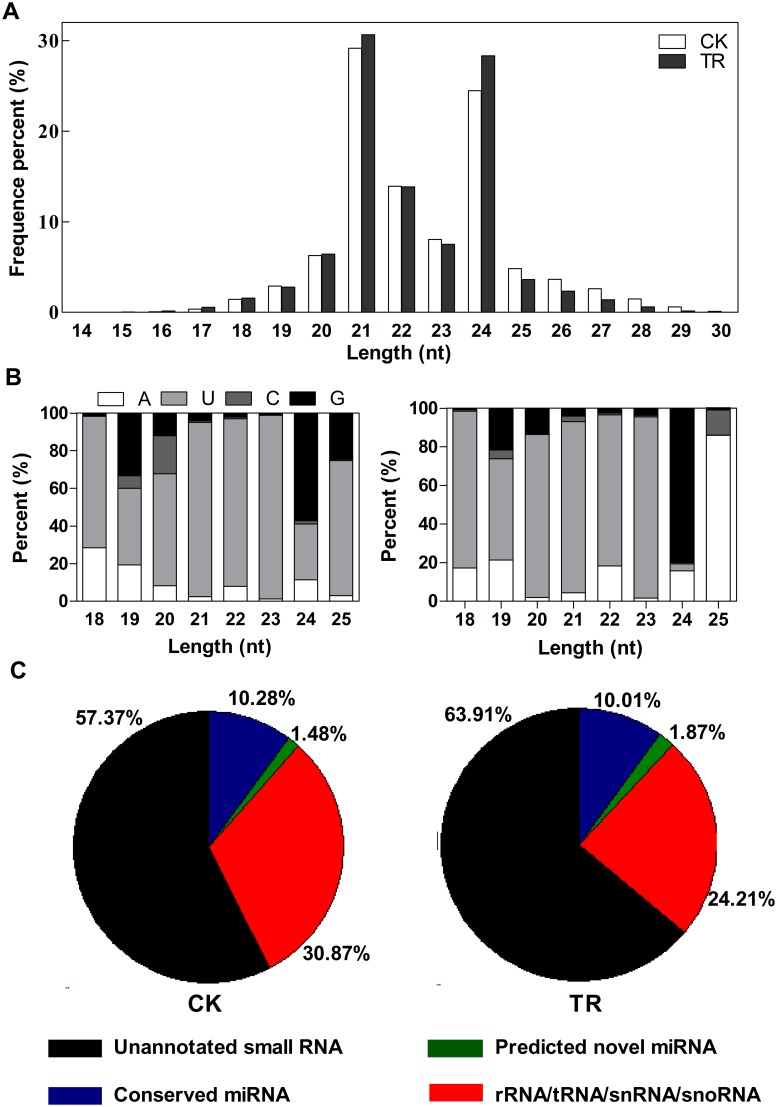
Length distribution (A), first nucleotide bias (B) and composition (C) of the small RNA in CK and TR libraries.

To survey small RNAs in *Paeonia ostii* and their role in plant response to copper stress, two small RNA libraries, with (TR) and without (CK) copper-treatment, were sequenced by Illumina sequencing technology. We obtained 7,655,306 reads from the CK library and 5,990,653 reads from the TR library after discarding adaptor sequences, filtering out low quality reads and cleaning up the contamination formed by the adaptor-adaptor ligation. The majority of the obtained sRNA sequences were 20–24 nt in the two libraries, which is within the typical size range for Dicer-derived products (Fig. 1A). As shown in Fig. 1, the most abundant classes were represented by 21- and 24-nt-long sRNAs, with an unexpected majority of the 21 nt, compared with 24 nt. In addition, analysis of the first nucleotide of 18–25 nt long sRNAs indicated that many sRNAs started with uridine (U) at their 5’-ends (Fig. 1B). rRNAs, tRNAs, snRNAs and snoRNAs were annotated by BLASTn to NCBI Genbank database and Rfam database (Fig. 1C, S1 Table). Among the reaming sequences, 786,809 sequences of CK library and 599,514 sequences of TR library were similar to known miRNAs from other plant species that had previously been deposited in miRBase 20.

### Conserved miRNAs and evolutionary conservation

Because above candidate miRNA sequences probably came from the same precursor as cleavage by RNase III enzymes was imprecise, these sequences were further clustered based on sequence similarity. We determined that the sequence with the dominant number of reads in a cluster was likely to be the real sequence due to its relatively high expression level, and these sequences were regarded as temporary miRNAs. Then, clean data was aligned to the above temporary miRNA database and the expression of miRNA is generated by summing the count of tags which can align to the temporary miRNA database within two mismatches. A total of 102 conserved miRNAs belonging to 89 families were identified in the two libraries (S2 Table). Overall, the miR157 family members were most abundant in *Paeonia ostii*. The results indicate that different members in some miRNA families displayed drastically different expression levels. For example, reads of members in miR157 ranging from 39 to 384,671 and 46 to 243,164 in CK and TR group respectively.

In order to analyze the evolutionary roles of the identified known miRNAs, we performed extensive comparisons against known miRNAs in other plant species (S3 Table). Although numerous plant miRNAs have been discovered and deposited in the public miRNA database, there is no available miRNA data of *Paeonia ostii* except 45 miRNAs of *Aquilegia caerulea* which belongs to *Ranunculaceae*. Among the identified known miRNAs of *Paeonia ostii*, eleven are highly evolutionarily conserved in plants from different divisions including magnoliophyta, coniferophyta and embryophyta division, suggesting that these miRNA play important and conserved roles in the plant kingdom. Twenty miRNAs are highly conserved in a variety of plant species from magnoliophyta division. For example, miR156, miR171, miR160, miR159, miR169, and miR167 have been found to have orthologs in more than 90% selected plant species. *Paeonia ostii* and other *Ranunculaceae* plant (*Aquilegia caerulea*) shared 18 conserved miRNA families.

### Identification of pre-miRNAs and *Paeonia ostii*-specific miRNA families

Given that no data were available to identify pre-miRNAs and *Paeonia ostii*-specific miRNA families, we performed transcriptome sequencing in *Paeonia ostii* with or without copper stress. To identify conserved and putative novel miRNA precursors sequences, the sRNA libraries were matched against the combined *Paeonia ostii* transcript sequences using the MIREAP pipeline. The candidate pre-miRNAs were predicted by exploring the secondary structure, the minimum folding free energy (MFE) and the minimum folding free energy index (MEFI). Our sequence analysis for all libraries showed that the putative pre-miRNAs of each library greatly varied from 67 to 370 nucleoties in length (S4 Table). The precursors had an average length of 142 bp, a CG content of 43.51%, an MFE of-49.975 which were similar to the pre-miRNA characteristics in other plant species.

**Fig 2:**
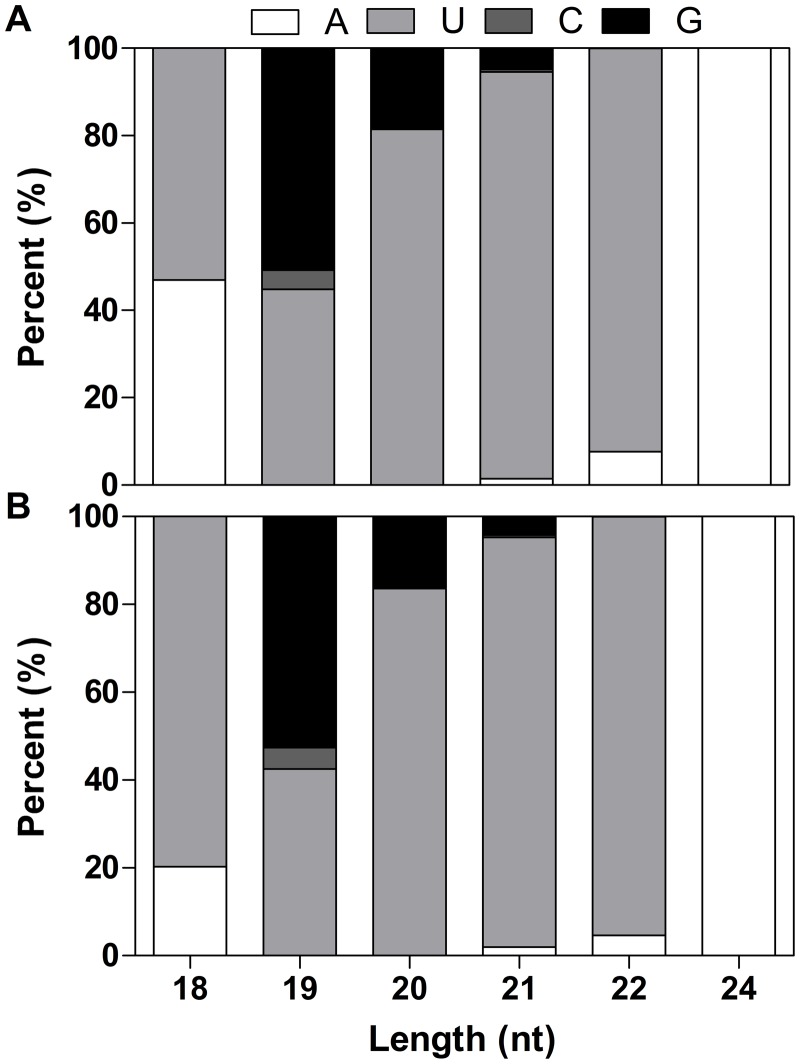
First nucleotide bias of novel miRNA in CK (A) and TR (B) libraries.

For 70 miRNA duplex-like pairs we predicted, 11 were identified as known full-length pre-miRNA sequences along with 6 miRNAs anchored in the 3p-arm and 5 miRNAs in the 5p-arm (S4 Table). In addition to conserved miRNAs, 59 sequences with characteristic hairpin-like structures were BLASTed against miRBase and NCBI databases, and no homologies with previously known plant miRNAs were found (S4 Table). These results revealed the existence of 34 novel miRNAs in *Paeonia ostii* (S4 Table). 17 miRNAs were anchored in the 3p-arm and 17 miRNAs in the 5p-arm of these pre-miRNAs. In agreement with previously observation for conserved miRNA, the majority of these newly determined novel miRNAs are 21nt longer and the uracil nucleotide is dominant in the first position of 5’ end (Fig. 2). The first nucleotide bias analysis showed that uracil was the most frequently used first nucleotide in miRNAs of *Paeonia ostii*. The most abundant novel miRNA yielded 66,943 reads, and it is the third most abundant miRNA in *Paeonia ostii*, suggesting an important role in this tissue.

### Identification of IsomiRNAs

Variants of miRNAs, also called isomiRNAs, are encoded by the same pre-miRNAs and exhibit sequence variations from the reference miRNAs in miRBase database. In the present study, the alignment of the sRNA library with identified *Paeonia ostii* pre-miRNAs allowed for the estimation of the number and abundance of each isomiRNA corresponding to conserved (S5 Table) and novel *Paeonia ostii* miRNA (S6 Table). The results showed that the length variations in abundant occurred predominantly in the 3’ end of the miRNAs, mainly in the form of missing nucleotides and/or terminal additions of nucleotides. In *Paeonia ostii* miRNA pools, the 5’ heterogeneity was common and only slightly less prevalent than 3’ heterogeneity. The conserved pre-miRNA mir157 and the novel pre-miRNA mir_15 produced more isomiRNAs than the other pre-miRNAs. Moreover, it was observed that some abundant sequences that could map to the identified pre-miRNAs of *Paeonia ostii* were not annotated as miRNA sequences in miRBase.

### Target prediction of miRNAs

To identify potential targets of *Paeonia ostii* miRNAs, we searched unigene sequences derived from high-throughput sequencing of *Paeonia ostii*. A total of 336 unigenes were predicted as potential targets of 64 known plant miRNAs and 27 novel miRNAs (S7 and S8 Tables). Most miRNAs have more than one predicted target, and some of the miRNAs even have more than 12. Additionally, for 45 miRNAs, no targets were predicted. It is possible that many of these miRNAs have unpredicted target RNAs or exist without actual targets.

For comprehensive annotation, all putative target transcripts in each library were analyzed by Gene Ontology (GO) terms (S1 Fig.). The conserved miRNA in two libraries have the same number of target genes. As shown in S1 Fig., more than one third target genes were involved in biological process, with the majority of conserved and novel miRNA targets localized in cellular process. In biological processes, 44 genes primarily participate in stimulus responses and different cellular process. We also identified that a significant number of the predicted targets were poorly characterized gene, suggesting possible new roles for these miRNAs in *Paeonia ostii*.

### Different expression profiles of small RNAs in CK library and TR library

**Fig 3:**
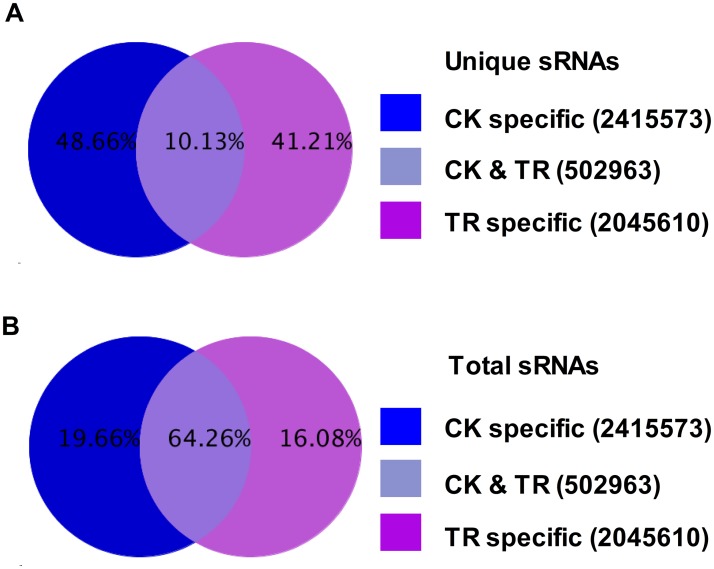
Common and specific sequences between CK and TR lirbary.

To detect the miRNAs which were responsive to copper stress, we summarized the common and specific sequences between two libraries of *Paeonia ostii*. The CK library contained more non-coding RNAs than TR library, which may indicate that *Paeonia ostii* induced more target gene generation under copper stress (Fig. 1). CK and TR library shared 8,769,236 (64.23%) sequences among the total sRNAs representing 502,963 (10.13%) unique sRNAs, which indicated that the sequences present in both libraries expressed higher than library-specific sequences (Fig. 3). In these unique sRNAs, the count of TR-specific sRNA was 2,045,610 (41.21%), which is small than CK-specific sRNAs (2,415,573 reads, 48.66%). These library-specific sRNAs showed responses to copper treatment.

The two libraries were then compared for their size distribution of small RNAs. In both libraries, the 21–24 nt RNAs represented the predominant species (about 76%), with 21-nt and 24-nt RNAs being the two most abundant classes (Fig. 1), consistent with the distribution patterns of small RNAs from other plant species. Under copper stress, the content of 21-nt and 24-nt RNAs were induced while most of other small RNAs were decreased. This observation suggests a more extensive regulation of gene expression by sRNAs at the transcriptional level in TR rather than in CK.

**Fig 4:**
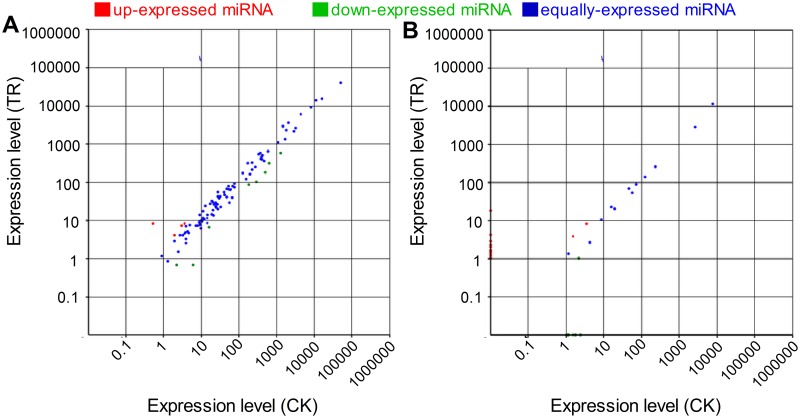
Scatter-plot graphs represent the conserved miRNA (A) and novel miRNA (B) differential expression patterns between control (CK) and copper stress (TR). The X axis indicates normalized gene expression levels in control and the Y axis indicates the normalized gene expression levels (per transcript) in copper-stresses tissues. The dots which are located at the upper and lower side of the diagonal line reflects the changes in the expression levels of miRNA genes; above the diagonal line, indicating up-regulation whereas below the diagonal line indicating down-regulation. For miRNA deep-sequencing experiment, the fold change cut-off was set at 1.5.

**Fig 5:**
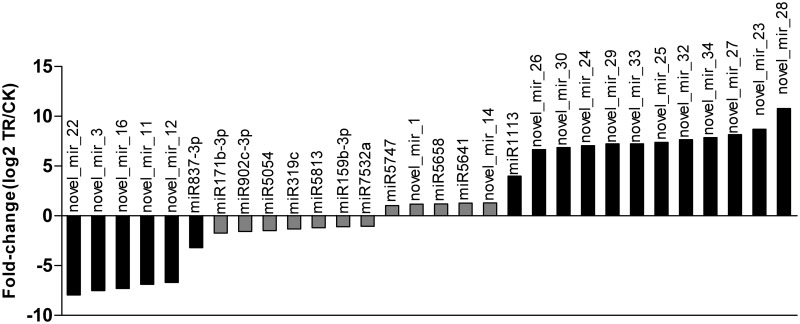
The differentially expression of significant changed miRNAs between CK and TR libraries.

We then made a comparative analysis of miRNA expression between the two libraries. The expression of miRNA in two libraries was shown by plotting Log2-ratio figure (Fig. 4) and detailed in S9 Table. From the two data sets, plenty of miRNAs were found to be differentially expressed between the two libraries. Overall, 59 miRNAs were down regulated and 74 were up regulated after copper treatment, which indicated that more genes were suppressed during copper treatment. Among them, 12 conserved miRNA and 18 novel miRNA showed significantly changed expression level in the copper-stressed *Paeonia ostii* seedlings (Fig. 5). miR1113, novel_mir_26, novel_mir_30, novel_mir_24, novel_mir_29, novel_mir_33, novel_mir_25, novel_mir_32, novel_mir_34, novel_mir_27, novel_mir_23 and novel_mir_28 were clearly up regulated (>3), whereas miR837–3p, novel_mir_12, novel_mir_11, novel_mir_16, novel_mir_3 and novel_mir_22 were down regulated. Among all miRNA genes, novel_mir_28 and novel_mir_22 showed the highest degree of induction and depression respectively.

It is also important to note that although some previous study showed that miR398, miR397, miR408 and miR857 are involved in plant copper response, in our study, these miRNAs were only slightly changed. In contrast, many novel miRNA identified in *Paeonia ostii* showed significantly changes in response to copper stress.

### Validation and expression patterns of miRNAs and their target genes

To confirm the results obtained from sRNA deep sequencing, the expression patterns of 9 copper-responsive miRNAs (fold changes>5) including miR902c-3p, novel_mir_3, novel_mir_14, novel_mir_22, novel_mir_23, novel_mir_27, novel_mir_32, novel_mir_33 and novel_mir_34 were validated by qRT-PCR (S10 Table). As expected, qRT-PCR results of nine selected miRNAs were similar in magnitude to those obtained by deep sequencing, and confirmed the changes in miRNA expression in response to copper stress. For conserved miRNAs, miR902c-3p was down-regulated upon copper stress. For novel miRNAs, only the expression of novel_mir_3 and novel_mir_22 decreased after treated with copper while most selected novel miRNAs were up-regulated.

We also examined the expression patterns of 16 corresponding target genes in our transcriptome sequencing data to evaluate if the observed differential expression in miRNA had a direct effect on their target transcript abundance. As shown in S10 Table, 16 target genes showed a clear opposite expression pattern compared with their corresponding miRNAs which suggest that miRNA-mediated regulation of target gene expression level appears to be occurring.

## Discussion

### The conserved and novel miRNAs in *Paeonia ostii*

MiRNAs are a class of small RNA molecules that has recently emerged as key regulator of gene activity. Identification of miRNAs and their targets is the basis for understanding the physiological functions of miRNAs. However, to our knowledge, no studies have been done on identifying and analyzing miRNAs in *Paeonia ostii*. In this study, we adapt high-throughput DNA sequencing to the discovery of endogenous small RNAs from *Paeonia ostii* and their response to copper stress was also analyzed. This work will provide useful information to deepen our understanding of the function and regulatory mechanisms of miRNAs in copper stress response. The high-throughput sequencing results showed that sRNAs of 21 nt and 24 nt in *Paeonia ostii* formed two major classes, occupying 38.35% and 32.06% (Fig. 1) of the total, respectively. The 21-nt class of sRNAs showed the highest abundance in both libraries instead of 24-nt reported in previous studies [35,36]. This observation is in agreement with some previous reports in grapevine and tomato [37,38] but contrasts to those reported in Arabidopsis, rice and peanut [39–42] which indicated that some differences might exist in the sRNA biogenesis pathways in various plants. A relatively small number of non-redundant sequences are expressed at a high level may also contribute to this difference.

Our data revealed the existence of 89 known miRNA families as well as 34 new predicted miRNAs in *Paeonia ostii* (S2 and S4 Tables). By extensive comparisons against known miRNAs in other plant species, 11 miRNA families were found in more than 20 plant species, and they were conserved between dicots and monocots, as well as in coniferophyta and embryophyta division (S3 Table). Plenty of previous studies showed that well conserved miRNAs (eg. miR156, miR159 and miR396) often retain homologous target interactions and perform analogous molecular functions across phyla in the long process of evolution. Thus, we assumed that these evolutionarily conserved miRNAs regulate target involved in many key metabolic processes of plants, and could be mobilized towards adaptive responses to adverse circumstances.

Based on their phylogenetic distribution, 89 conserved families identified in *Paeonia ostii* could be classified into 4 groups. Ten families (miR171, miR156, miR159, miR160, miR166, miR167, miR408, miR169, miR319, miR396, miR390,) are present in magnoliophyta, coniferophyta and embryophyta; 6 families are present in magnoliophyta and coniferophyta, but not embryophyta; 8 families are restricted to magnoliophyta and embryophyta; and the remaining 65 families are magnoliophyta-specific. Different conserved miRNAs in *Paeonia ostii* indicated that some of them are from ancient families and some appear to be much younger (for example, *Paeonia ostii*-specific miRNA families). These age differences suggested that there is an ongoing process of miRNA evolution. Previous work in Arabidopsis has also indicated that the birth and death of miRNA families is a common phenomenon in plant evolution [43].

To find out *Paeonia ostii*-specific miRNA families, we integrated the data from *Paeonia ostii* small RNA libraries with its EST database which however, did not provide any significant findings, because of the low coverage of *Paeonia ostii* EST database. Thus, we performed transcriptome sequencing in *Paeonia ostii* with or without copper stress. Finally, 34 sRNA sequences derived from *Paeonia ostii* transcriptome sequencing data with intra molecular folding capacities and not previously described as miRNAs in other plant species were predicted as potential *Paeonia ostii* specific miRNAs (S4 Table). In contrast with common conserved miRNAs, the predicted novel miRNAs are often expressed at a very low level, which is consistent with reports suggesting that species-specific miRNAs are usually expressed at low level [35]. Of the 34 putative miRNAs in both libraries, only five miRNAs (novel_mir_5, novel_mir_6, novel_mir_7, novel_mir_9 and novel_mir_15) were sequenced more than 500 times.

Because of the inaccuracies in Dicer pre-miRNA processing, pre-miRNA could generate some sequence variants that show additional nucleotides in the 5’ or 3’ terminus compared to the canonical or most abundant mature miRNAs. In the process of detecting known miRNAs in *Paeonia ostii*, we found that in some cases, the most abundant sequences among all unique sequences mapped to the identified pre-miRNAs of *Paeonia ostii* were not annotated as miRNA sequences in miRBase (S5 Table). Numerous recent studies suggest that at least some variants may affect target selection, miRNA stability, or loading into the RNA-induced silencing complex (RISC) [44]. It was also observed that miRNA families differ significantly from each other in the number and abundance of isomiRNAs, as was observed in other studies [45]. This wide variation suggests that different members in the same miRNA family might involve in divergent functions and might be necessary at different levels or even expressed in a cell-specific manner, according to the species, timing, tissue and/or other situations.

### The copper-responsive miRNA and their targets in *Paeonia ostii*

Heavy metal pollution is an increasing environmental problem worldwide. Recently, miRNAs have emerged as important modulators of plants adaptive response to heavy metal stress. It is reported that plant heavy metal resistance gene families are comprised of hundreds of members, which are usually targeted by sRNAs. Since little sRNA information is available for *Paeonia ostii*, a global survey of sRNAs in *Paeonia ostii* seedlings with and without copper treatment will facilitate our understanding of the regulatory mechanisms of *Paeonia ostii* in response to copper stress, which would provide useful information for improving the heavy mental resistance of *Paeonia ostii* and other economically important plants.

In this study, a number of miRNAs have been identified to be differentially expressed in *Paeonia ostii* under copper treatment (Fig. 5, S9 Table). Compared with CK, 5 novel miRNA genes including novel_mir_3, 11, 12, 16, and 22, were expressed exclusively in CK lirbrary. On the other hand, we found that 11 novel miRNA genes including novel_mir_23, 24, 25, 26, 27, 28, 29, 30, 32, 33, and 34 were TR-specific. Moreover, a total of 14 miRNAs detected in both libraries were found to exhibit significant expression changes. However, some previously reported miRNAs including miR398, miR397, miR408, and miR857 that are participated in plant copper stress response didn’t show significant changes in *Paeonia ostii* [2]. As a copper tolerant plant, *Paeonia ostii* might have different regulating mechanisms in coping with environment copper stress.

Target prediction of the differentially expressed miRNAs could provide information on the biological processes regulated miRNA. It has been shown that plant miRNAs exhibit high degree of sequence complementarity to their targets, which allows for target prediction [46]. The present study identified 27 transcript targets for 13 significantly changed miRNAs, nine of which are novel species-specific miRNAs from *Paeonia ostii* (S7 and S8 Tables). A proportion of the targets are shown regulate plant resistance to biotic and abiotic stresses. For example, miR902c targets several transcripts coding stress resistance gene including mitochondrial gene and metallocarboxypeptidase inhibitor gene. However, most target genes of novel miRNAs were novel and had unknown function. Most of these novel miRNAs down-regualted in response to copper stress (S7, S8 and S10 Tables). Further understanding of these novel miRNAs’ functions will be aided by detailed analysis of target genes. We concluded that these putative novel miRNAs are involved in regulation of *Paeonia ostii*-specific processes which may contribute to the strong tolerant of *Paeonia ostii* to copper stress. The study of the newly identified *Paeonia ostii* miRNAs and their target genes will provide us with tools to approach these issues.

## Supporting Information

S1 Fig Targets of the miRNAs identified in *Paeonia ostii*. The number of genes for each Gene Ontology (GO) term is relative to the total number of contigs from each gene category.
(TIF)

S1 Table Statistics of small RNA sequences from CK and TR libraries of the *Paeonia ostii*. (DOC)

S2 Table Conserved miRNAs in *Paeonia ostii*. (DOC)

S3 Table Conserved miRNA families in *Paeonia ostii* and across-species. Evolutionary conservation of 89 miRNA families identified in *Paeonia ostii* within plant species reported in miRBase (Version 20). Closely related species are shown in the same color. Colored boxes denote the presence of the indicated miRNA family.
(XLS)

S4 Table Predicted pre-miRNA of conserved and novel miRNAs in *Paeonia ostii*. (XLS)

S5 Table Conserved miRNA isoforms in CK and TR libraries. (XLS)

S6 Table Novel miRNA isoforms in CK and TR libraries. (XLS)

S7 Table Predicted targets of known miRNAs in *Paeonia ostii*. (XLS)

S8 Table Predicted targets of novel miRNAs in *Paeonia ostii*. (XLS)

S9 Table Differential expression of identified known and novel miRNAs between libraries. (XLS)

S10 Table Validation of miRNAs and their target genes. (XLS)
