# Supplementary figures and images for: Identification and Characterization of MicroRNAs from Tree Peony (Paeonia ostii) and Their Response to Copper Stress
Source: PLoS One. 2015 Feb 6;10(2):e0117584. doi: 10.1371/journal.pone.0117584 (PMC4319853; doi:10.1371/journal.pone.0117584)

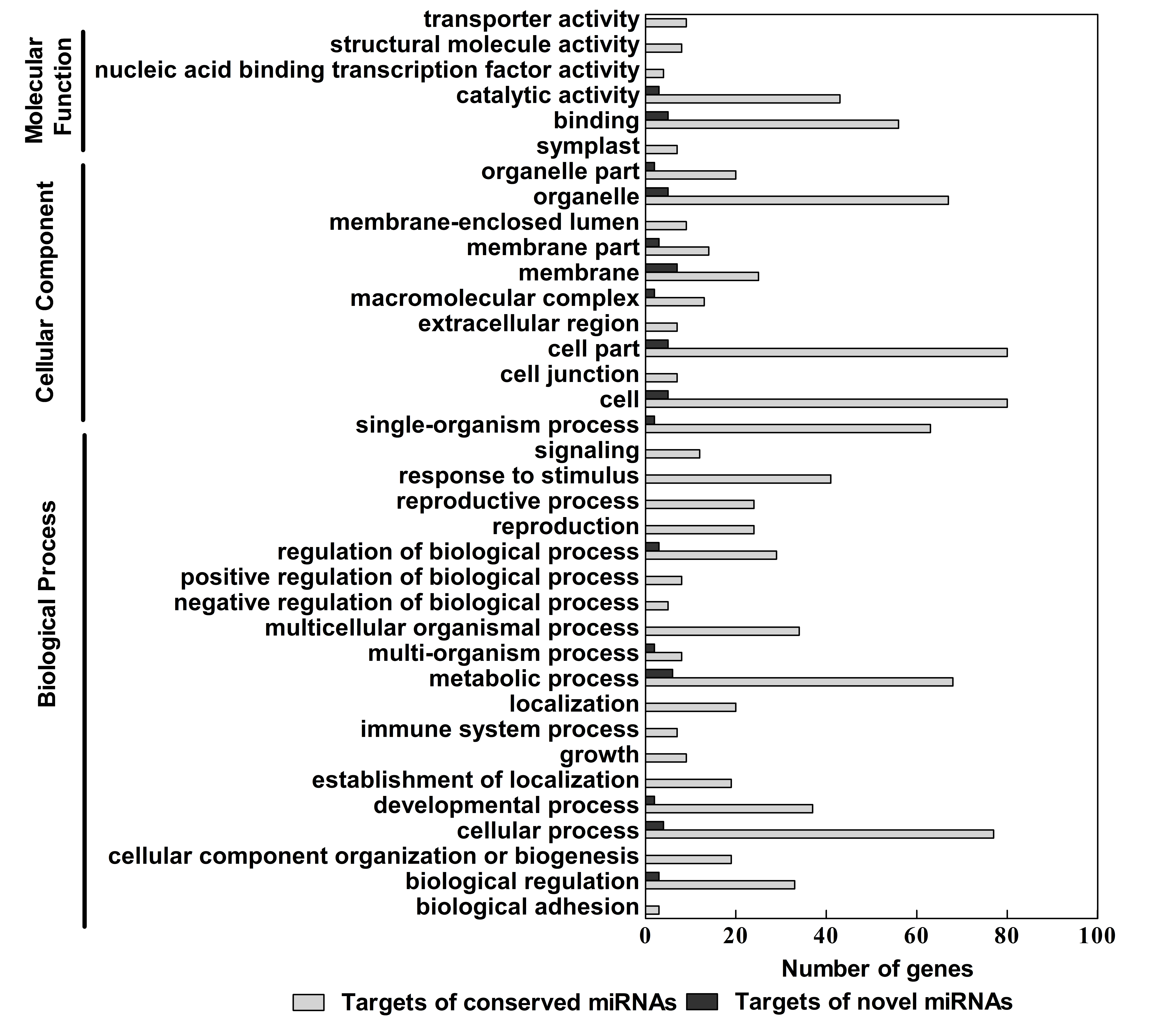

Supplement: S1 Fig — The number of genes for each Gene Ontology (GO) term is relative to the total number of contigs from each gene category. (TIF) [file pone.0117584.s001.tif]
