# Supplementary material for: Identification and Characterization of MicroRNAs from Tree Peony (Paeonia ostii) and Their Response to Copper Stress
Source: PLoS One. 2015 Feb 6;10(2):e0117584. doi: 10.1371/journal.pone.0117584 (PMC4319853; doi:10.1371/journal.pone.0117584)
Supplement: S1 Table — (DOC) [file pone.0117584.s002.doc]

**Table S1.** Statistics of small RNA sequences from CK and TR libraries of the *Paeonia ostii*

|  | Total reads | Percent (%) | Unique reads | Percent (%) |
| --- | --- | --- | --- | --- |
| CK |  |  |  |  |
| Raw reads | 7,733,041 |  |  |  |
| Clean reads (18-30 nt sRNA) | 7,655,306 | 100% | 2,918,536 | 100% |
| miRNA | 786,809 | 10.28% | 15,296 | 0.52% |
| Predicted novel miRNA | 113,081 | 1.48% | 381 | 0.01% |
| rRNA/tRNA/snRNA/snoRNA | 2,363,362 | 30.87% | 318,918 | 10.92% |
| Un-annotation | 4,392,045 | 57.37% | 2,583,941 | 88.86% |
| TR |  |  |  |  |
| Raw reads | 6,070,261 |  |  |  |
| Clean reads (18-30 nt sRNA) | 5,990,653 | 100% | 2,548,573 | 100% |
| miRNA | 599,514 | 10.01% | 13,073 | 0.51% |
| Predicted novel miRNA | 111,787 | 1.87% | 447 | 0.02% |
| rRNA/tRNA/snRNA/snoRNA | 1,450,727 | 24.21% | 217,775 | 8.54% |
| Un-annotation | 3,828,625 | 63.91% | 2,317,278 | 90.95% |
