# Supplementary material for: Identification and Characterization of MicroRNAs from Tree Peony (Paeonia ostii) and Their Response to Copper Stress
Source: PLoS One. 2015 Feb 6;10(2):e0117584. doi: 10.1371/journal.pone.0117584 (PMC4319853; doi:10.1371/journal.pone.0117584)
Supplement: S2 Table — (DOC) [file pone.0117584.s003.doc]

**Table S2.** Conserved miRNAs in *Paeonia ostii*

| **miRNA family** | **miRNA name** | **Sequence in the tree peony (5’-3’)** | **CK** | **TR** |
| --- | --- | --- | --- | --- |
| miR156 | miR156a | TGACAGAAGAGAGTGAGCAC | 86666 | 81782 |
|  | miR156f-3p | GCTCACCCTCTATCTGTCACC | 193 | 131 |
| miR157 | miR157a | TTGACAGAAGATAGAGAGCAC | 384671 | 243164 |
|  | miR157c-3p | GCTCTCTATACTTCTGTCATCC | 39 | 46 |
| miR159 | miR159b | TTGCATAACTCAGGAGCTGC | 4545 | 3821 |
|  | miR159b-3p | ATTGGAGTGAAGGGAGCTCCC | 1393 | 510 |
| miR160 | miR160a | TGCCTGGCTCCCTGTATGCCA | 111 | 68 |
|  | miR160a-3p | GCGTATGAGGAGCCAAGCATA | 215 | 116 |
| miR162 | miR162a | TCGATAAACCTCTGCATCCAG | 81 | 57 |
| miR164 | miR164a | TGGAGAAGCAGGGCACGTGCA | 986 | 1016 |
|  | miR164c-3p | CATGTGCCCTTCTTTCCCATC | 7 | 5 |
| miR165 | miR165a | TCGGACCAGGCTTCATCCCCC | 78 | 58 |
| miR166 | miR166a | TCGGACCAGGCTTCATTCCCC | 63523 | 56088 |
|  | miR166g-3p | TCGGACCAGGCTTCATTCCTC | 15845 | 21426 |
| miR167 | miR167a | TGAAGCTGCCAGCATGATCTGA | 34021 | 35910 |
|  | miR167f-3p | AGATCATGTGGCAGTTTCACC | 61 | 44 |
| miR168 | miR168a | TCGCTTGGTGCAGGTCGGGAA | 11733 | 17184 |
|  | miR168a-3p | CCCGCCTTGCATCAACTGAAT | 425 | 173 |
| miR169 | miR169b | CAGCCAAGGATGACTTGCCGG | 195 | 164 |
|  | miR169j-3p | TGGTGAAGCCTTCGTGACGG | 2955 | 2400 |
| miR170 | miR170-5p | TATTGGCCCGGTTCATTCAGA | 7 | 7 |
| miR171 | miR171a | TGATTGAGCCGCGCCAGTATC | 73 | 48 |
|  | miR171b-3p | TTGAGCCGCGTCAATATCTCT | 17 | 4 |
| miR172 | miR172a | AGAATCTTGATGATGCTGCAT | 521 | 236 |
| miR319 | miR319c | TGGAGTGAAGTGAGCTCCTTA | 127 | 40 |
| miR390 | miR390a-3p | CGCTATCCATCCTGAGTCTCA | 31 | 19 |
|  | miR390b-5p | AAGCTCAGGAGGGATAGCACC | 2644 | 3256 |
| miR393 | miR393a-3p | ATCATGCTATCCCTTTGGATT | 15 | 17 |
| miR394 | miR394a | TTGGCATTCTGTCCACCTCC | 28 | 27 |
| miR395 | miR395x | GTGAAGTGTTCGGATCGCGTC | 13218 | 8017 |
| miR396 | miR396a | TTCCACAGCTTTCTTGAACTG | 256 | 135 |
|  | miR396b-3p | GCTCAAGAAAGCTGTGGGAAA | 152 | 195 |
| miR399 | miR399c-5p | GGGTAGACTCCTTTGCAGA | 24984 | 15656 |
| miR403 | miR403 | TTAGATTCACGCACAAACCCG | 34 | 31 |
| miR408 | miR408-5p | ACAGAGGAAATGCAGAGCCAG | 158 | 180 |
| miR415 | miR415 | GCAGAACAGAAGGCAGAACAG | 3273 | 3037 |
| miR477 | miR477c | TTCCTCGAAGGCTTCCAATAT | 148 | 85 |
| miR479 | miR479 | TGTGATGTTGGTTCGGCTCATC | 113 | 51 |
| miR482 | miR482d-5p | GGATGGGTGGCTGAGACGG | 216 | 326 |
| miR529 | miR529 | TGAAGAAGAGATAGTAGTAGAG | 249 | 254 |
| miR535 | miR535d | TGACGGCGAGAGAGAGCACAC | 1339 | 1893 |
| miR774 | miR774b-5p | TGAGATGATGATTTGGATA | 56 | 44 |
| miR827 | miR827 | TTAGATGACCATCAACGAACA | 36 | 28 |
| miR833 | miR833a-5p | TTTTTCGTCGTACTCGGCTAGT | 600 | 434 |
| miR837 | miR837-3p | AAAGAACAAAAAGCCGGATGG | 47 | 4 |
| miR849 | miR849 | TAGCGAAATCATGGTGTAGTA | 110 | 83 |
| miR894 | miR894 | GTTTCACGTCGGGTTCACCA | 14055 | 14062 |
| miR902 | miR902c-3p | ACGAAAGTCGGTCATAGT | 2282 | 604 |
| miR1077 | miR1077-5p | TTGAAGTGTTCGGATCGCGGC | 8690 | 6561 |
| miR1113 | miR1113 | TGAGCAGATCTTGGGTAGCCT | 4 | 50 |
| miR1156 | miR1156.2 | TCAGCTGGAACTTTACGGCAC | 233 | 230 |
| miR1223 | miR1223a | TTGAGCAGTCAGAGACCTCCA | 21 | 24 |
| miR1310 | miR1310 | AGGCATCGGGGGCGCAACGCC | 361 | 246 |
| miR1514 | miR1514a | TTCATTCTGTATAATAGGCTT | 85 | 104 |
| miR1520 | miR1520m | AATCAGAACAGAGATGGACATT | 3143 | 2730 |
| miR1861 | miR1861a | TGATCTTGAGGAGCAAGCTGAT | 190 | 172 |
| miR2109 | miR2109 | TGCGCGTATCTTCGCCTCTGA | 534 | 471 |
| miR2111 | miR2111a-5p | TAATCTGCATCCTGAGGTTTA | 31 | 15 |
| miR2199 | miR2199 | TGATAACTCGACGGATCGC | 22235 | 12871 |
| miR2868 | miR2868 | CTTGTTTTTGGTGTAGTAGGAA | 25 | 24 |
| miR2916 | miR2916 | GGGGCTCGAAGACGATCAGAT | 3165 | 2353 |
| miR3442 | miR3442-3p | TTCAGAGTTAAGAACATGGTT | 116 | 81 |
| miR3627 | miR3627-5p | TTGTCGCAGGAGTGATGGCACT | 479 | 389 |
| miR3630 | miR3630-5p | GCAAGTGACGATAAACAGACA | 76 | 37 |
| miR3933 | miR3933 | ACAAGCAAATGACGCACTCTG | 363 | 399 |
| miR4414 | miR4414a-3p | ATCCAACGATGCAGGAGCTGG | 1703 | 1939 |
|  | miR4414b | TGTGAATGATGCGGGAGACAA | 135 | 162 |
| miR5054 | miR5054 | GTTCCCCACAGACGGCGCCA | 3837 | 1074 |
| miR5059 | miR5059 | TCGTTCCTGGGCAGCAACACCA | 1520 | 975 |
| miR5072 | miR5072 | CGTTCCCCAGCAGAGTCGCCA | 1206 | 729 |
| miR5077 | miR5077 | TTCACGTCGGGTTCACCA | 2938 | 3445 |
| miR5083 | miR5083 | AGACTACAATTATCTGATCA | 19 | 9 |
| miR5139 | miR5139 | AACCTGGCTCTGATACCA | 321 | 273 |
| miR5205 | miR5205a | CTTAGAATTTGGACGAGGGAG | 314 | 321 |
| miR5213 | miR5213-5p | TGCGTGTGTCTTCACCTCTGA | 1706 | 1261 |
| miR5230 | miR5230 | CAAATCTGAATCCATACGGCA | 208 | 157 |
| miR5241 | miR5241a | TGATGAATGGAAGACTGCTTT | 187 | 139 |
| miR5248 | miR5248 | TTTTTATAGGCATGCATTTCT | 71 | 46 |
| miR5485 | miR5485 | TGACAGATTGGTATCAGAGCA | 214 | 253 |
| miR5641 | miR5641 | TGGATAGAAGATGAAGAGTTG | 23 | 44 |
| miR5642 | miR5642a | TCTCGCACTGTATAACGGCTTT | 87 | 67 |
| miR5658 | miR5658 | ATATGGTGTTGATGATGATAA | 28 | 50 |
| miR5671 | miR5671 | CATGGTGGTGACGGGTGAC | 2156 | 1483 |
| miR5675 | miR5675 | TAGAAACGGACACAATGGTAA | 10 | 5 |
| miR5747 | miR5747 | AGAAGAAGCTCATACATGAACATT | 15 | 24 |
| miR5769 | miR5769 | TGAGGGAAAGAAGACAAAGAA | 98 | 144 |
| miR5782 | miR5782 | AAGCGGTAGGAGGACGTTCAG | 406 | 463 |
| miR5813 | miR5813 | ACAGCAGGACGGTGGTCATGGA | 10211 | 3463 |
| miR5827 | miR5827 | TTTATTGCGATTTGGACACTC | 162 | 111 |
| miR6113 | miR6113 | TCTGAAACTCAAGAAAACGTCG | 124364 | 89459 |
| miR6300 | miR6300 | GTCGTTGTAGTATAGTGGTA | 3597 | 2123 |
| miR6430 | miR6430 | TGATGGATTAATTGTTGCATA | 92 | 62 |
| miR6432 | miR6432 | TCGGGTCTAGAATAAAGATGG | 170 | 156 |
| miR6464 | miR6464 | TGATAGCTTTGTTGGATGTTA | 70 | 81 |
| miR6478 | miR6478 | CCGACCTTAGCTCAGTTGGTA | 466 | 368 |
| miR6483 | miR6483 | ATTGTAGAAATTTTCGGTATC | 69 | 53 |
| miR7127 | miR7127a | ATACTCAATCCAATTCTCATA | 31 | 43 |
| miR7532 | miR7532a | GAACAGCCTCTGGTCGATGGA | 4920 | 1853 |
| miR7536 | miR7536a | TAAGACATACTCGCAAGATTG | 30 | 29 |
| miR7722 | miR7722-3p | TAAGGGTACGAGGATGAGAAG | 412 | 389 |
| miR7753 | miR7753-3p | TCAGCAAGGGTAGAAGGCATG | 598 | 545 |
| miR8155 | miR8155 | CGTAACCTGGCTCTGATACCA | 165 | 79 |
